# Similar PTSD symptom networks observed in male and female survivors of military sexual assault: implications for understanding trauma responses

**DOI:** 10.3389/fpsyg.2024.1452417

**Published:** 2024-11-07

**Authors:** Bingyu Xu, Rebecca K. Blais, Rick A. Cruz, Hallie S. Tannahill

**Affiliations:** ^1^Department of Psychology, Arizona State University, Tempe, AZ, United States; ^2^Sheppard Air Force Base, Wichita Falls, TX, United States

**Keywords:** PTSD, military sexual assault, network analysis, sex differences, veterans

## Abstract

Posttraumatic stress disorder (PTSD) is a heterogeneous disorder with no universal symptom presentation. Sex differences in rates of PTSD among military samples are established, such that females are more likely to be diagnosed with PTSD, with severity particularly heightened among females exposed to military sexual assault (MSA). However, limited research has examined the PTSD symptom network structure among MSA survivors and whether it differs by sex. The current study examined global and sex-specific PTSD symptom network structure of PTSD among veterans and service members who were exposed to MSA. Participants were 400 service members/veterans (54% active duty; 50% male) with a history of MSA exposure recruited through Qualtrics. Participants completed an online survey assessing PTSD symptoms. Network analysis was conducted for the full sample to examine the overall symptom structure. Centrality indices revealed apathy to be the most central symptom, followed by irritability, hyperarousal, hypervigilance, and external avoidance. The Network Comparison Test was utilized to examine potential sex differences in network structure and global strength. There were no sex differences in global structure or network strength. Core symptom network structures of PTSD may be similar for males and females following MSA. Though males and females experience notable differences in PTSD, network structure is not among them. Central symptoms, or the potential treatment targets, could be similar for males and females MSA survivors.

## Introduction

There is a notable sex disparity in the development of posttraumatic stress disorder (PTSD) following exposure to trauma, with females being twice as likely as males to develop the disorder (Kilpatrick et al., 2013; Pineles et al., 2017). While differences in patterns of trauma exposure cannot fully explain this sex difference (Tolin and Foa, 2006; Olff et al., 2007), multiple studies have consistently shown that females experience higher levels of PTSD symptoms across various trauma types, including earthquakes (Carmassi and Dell’Osso, 2016), motor vehicle accidents (Fullerton et al., 2001), combat exposure (Kline et al., 2014; Luxton et al., 2010), and terrorism (Sever et al., 2008; Solomon et al., 2005). Such sex differences are also particularly notable among military sexual trauma survivors.

Military sexual trauma (MST) includes any instance of unwanted sexual attention (i.e., sexual harassment), attempted sexual assault, or sexual assault that was perpetrated during military service. Most studies combine both harassment-only and MST that includes assault, or military sexual assault (MSA). Overall, MST among females is associated with higher risk for PTSD compared to males (Kimerling et al., 2007; Kimerling et al., 2010; Maguen et al., 2012). However, these effects can be even stronger among females specifically reporting MSA (Tannahill et al., 2020; Tannahill and Blais, 2023). Established sex differences in psychobiological stress response systems, including the Hypothalamic–Pituitary–Adrenal axis (Olff et al., 2007; Verma et al., 2011), further support the notion that sex differences play a role in the development and maintenance of PTSD. It is therefore critical to better understand how PTSD symptomatology may vary as a function of biological sex.

One way to build on the extant literature on sex differences in PTSD development and maintenance is to apply novel symptom network approaches. Unlike latent variable theories, which suggest that symptoms originate from a common cause (Borsboom and Cramer, 2013), symptom network theory shifts the focus from attempting to model unobserved constructs to the interconnections between observed variables (i.e., symptoms). PTSD is characterized by a range of symptoms grouped into several clusters: re-experiencing, avoidance, negative alterations in cognition and mood, and alterations in arousal and reactivity. Network analysis helps in identifying how these symptoms influence one another within and across these clusters. Symptom networks are commonly analyzed using graphical models. These models provide insights into the pattern of intercorrelations between symptoms (“edges”), often yielding clusters or communities of symptoms. Centrality indices provide various ways of quantifying the importance of symptoms (“nodes”). The network theory of mental health concerns considers symptoms characterizing a clinical syndrome as potentially mutually influencing entities that can be modeled as a network structure. This approach can provide valuable insights into understanding how symptoms relate to one another and contribute to the overall manifestation and maintenance of a specific mental disorder (Borsboom, 2017; Fried and Cramer, 2017). In addition, network analysis can identify symptoms that have strong connections to other symptoms and are hypothetically influential in driving the overall symptomatology. These central symptoms are considered key nodes in the network, or potential mechanisms that maintain symptom profiles, and it is hypothesized that targeting these symptoms in interventions may have a more significant impact on the entire symptom complex (Fried et al., 2017).

The use of network analysis to better understand PTSD symptom profiles is growing. Meta-analysis has demonstrated that there is not a single symptom that plays the most central role in the PTSD network, although detachment, intrusions, and physiological reactivity are consistently highlighted as some of the most central symptoms (Isvoranu et al., 2021). This meta-analysis of PTSD symptom networks across 52 different samples observed large between-sample heterogeneity, indicating differential symptom networks for different populations and trauma types (Isvoranu et al., 2021). The 2021 meta-analysis suggested that, in order to address the large between-sample heterogeneity, future research should focus on specific types of trauma and homogeneous samples when constructing a PTSD network structure. To our knowledge, only one study conducted by Xu et al. (2024) has investigated the network structure of PTSD symptoms in relation to MST. According to results from Xu et al. (2024), we hypothesized that the PTSD network structure in our sample follows the same pattern, with detachment, psychological/physiological reactivity, intrusive thoughts, and internal avoidance among the most central symptoms.

We found no prior study that had explored potential sex differences in PTSD network structure associated specifically with MSA. Understanding the network structure of PTSD among MSA survivors and whether it differs by sex could provide additional insights into symptom structures that are relevant for treatment following MSA. Given the limited literature investigating sex differences among MSA survivors, the current study pursued an exploratory aim of examining potential sex differences in the symptom network structure.

## Methods

The current study used existing data from a parent study that examined individual and interpersonal outcomes of MSA among service members and veterans (Tannahill et al., 2021). The parent study examined the association of posttraumatic cognitions, sex, and PTSD severity, making the current study a novel use of the dataset. Participants were recruited via Qualtrics. Inc. (Qualtrics Panels, 2017) during 2021. Inclusion criteria were post-9/11 era service members/veterans aged 18–65 who spoke English and were exposed to MSA. History of MSA was screened by asking (1) ‘When you were in the military, did someone ever have sexual contact with you against your will or when you were unable to say no (for example, after being forced or threatened, or to avoid other consequences)?’ and (2) ‘When you were in the military did someone try to have sexual contact with you against your will or when you were unable to say no?’ Answering ‘yes’ to either of the questions indicated a history of MSA. The parent study was approved by the Institutional Review Board at Utah State University (#11465; approved on 05/11/2021). The current secondary analysis was approved by the Institutional Review Board at Arizona State University (STUDY00016630; approved on 09/26/2022).

### Participants

Participants were 400 U.S. service members/veterans (50.00% female sex and 50.00% male sex) with a history of MSA, with an average age of 35.89 years (SD = 5.65). Most participants were White (*n* = 286, 71.50%) and had served or served in the Army (*n* = 292, 73.00%). Around half of the participants had been discharged (*n* = 184, 46.00%), and held a rank of Officer (*n* = 224, 56.00%). Preliminary descriptive statistics indicate that males were more like to be White, an Officer, and actively serving, compared to females. Table 1 presents the descriptive statistics of the sample.

**Table 1:** Sample descriptive statistics, stratified by sex (*N* = 400).

	Females *n* = 200 (50%)	Males *n* = 200 (50%)		
Variable	*n (%)*/M (SD)		*χ*^2^ test/*t*-test	Phi/Cohen’s *d*
Age	35.88 (5.93)	35.90 (5.38)	*t* (398) = −0.35	5.66
Marital status			*χ*^2^(1) = 0.77	0.04
Partnered	170 (85.00%)	176 (88.00%)		
Other	30 (15.00%)	24 (12.00%)		
Rank			*χ*^2^(1) = 13.15**	0.18
Enlisted	106 (53.00%)	70 (35.00%)		
Officer	94 (47.00%)	130 (65.00%)		
Discharge status			*χ*^2^(1) = 27.21**	−0.26
Service member	82 (41.00%)	134 (67.00%)		
Veteran	118 (59.00%)	66 (33.00%)		
Branch			*χ*^2^(1) = 5.20*	0.11
Army	138 (69.00%)	158 (79.00%)		
Non-army	62 (31.00%)	42 (21.00%)		
Race			*χ*^2^(1) = 2.81	0.08
White	137 (68.50%)	152 (76.00%)		
Non-white	63 (31.50%)	48 (24.00%)		
Sexual orientation			*χ*^2^(1) = 0.03	−0.01
Straight	180 (90.00%)	181 (90.50%)		
Sexual minority	20 (10.00%)	19 (9.50%)		
PTSD symptoms	46.99 (17.66)	49.91 (19.36)	*t* (394) = −1.57	18.53

PTSD, posttraumatic stress disorder. *p < 0.01; **p < 0.001.

## Measures

### PTSD symptoms

The *PTSD Checklist for DSM-5* (PCL-5; Weathers et al., 2013) was used to measure PTSD symptoms. The PCL-5 is a self-report scale with 20 items ranging from 0 (*not at all*) to 4 (*extremely*) regarding how much the symptom has been bothering the participant in the past month. Items are summed for a total score that ranges from 0 to 80 and higher scores indicate greater distress. Participants were instructed to respond to each item as it related to their MSA. The *PCL-5* showed excellent internal reliability in the current sample (Cronbach’s *α* = 0.96). The average score on the *PCL-5* in this sample was 48.46 (SD = 18.57), with 80.75% of the sample scoring above the clinical cutoff of 31, which suggests a probable PTSD diagnosis was present (Bovin et al., 2016). However, given the self-report nature of the data collection, additional assessments would be needed to verify the true presence of PTSD.

### Analytic plan

#### Network modeling

All analyses were performed using R version 4.3.0 (R Core Team, 2023). Network analysis was performed first for the full sample and then separately for males and females. In psychological networks, items are typically represented as “nodes” within the network, and the associations between nodes (partial correlations) are referred to as “edges.” When dealing with ordinal data, a penalized model based on the extended Bayesian Information Criteria (EBIC) is commonly used, as it allows for the input of partial covariance matrices to accommodate ordinal data (Epskamp et al., 2012, 2018). Given that symptoms assessed by the *PCL-5* are on an ordinal scale, a polychoric correlation matrix was computed by specifying the *cor_auto* function from the *qgraph* package. We employed the *EBICglasso* function from the *qgraph* package (Epskamp et al., 2012) to estimate the network structure. The selection of the EBIC tuning parameter gamma (*γ*) regulates the emphasis on model parsimony. Typically, *γ* is set at 0.25 or 0.50, with higher values indicating a preference for more parsimonious models. We specified *γ* = 0.5 as it strikes a more conservative balance between model complexity and parsimony, allowing for meaningful interpretation of the most important intercorrelations in the network structure (Epskamp et al., 2018). Lasso regularization minimizes the risk of spurious correlations and model overfitting by constraining small partial correlations to zero. We estimated edge weights and centrality indices using 1,000 non-parametric bootstrap samples to obtain stable and accurate estimates of edge weights and centrality indices, considering the inherent variability in the data. Edges were retained in the network only if they appeared in at least 75% of the bootstrapped samples. This criterion ensures greater robustness and reliability of the estimated network (Epskamp et al., 2018).

Centrality indices, such as Strength, Closeness, Betweenness, and Expected Influence, were computed to assess the importance of individual nodes within the network. *Strength* measures the overall connection strength of a node, reflecting the sum of its edge weights with other nodes (Opsahl et al., 2010). *Betweenness* quantifies the extent to which a node bridges connections between other nodes, highlighting its potential role in information flow or communication between different parts of the network (Opsahl et al., 2010). *Closeness* captures how close a node is to all other nodes in the network, indicating its ability to transmit information efficiently (Opsahl et al., 2010). *Expected Influence* estimates the influence of a node on the rest of the network, considering both direct connections and indirect pathways (Epskamp et al., 2018). A case-dropping bootstrap procedure was used to examine the stability of centrality measures by examining how robust these metrics were as smaller subsets of the sample were tested. A Case dropping Stability coefficient (CS coefficient) close to 1 indicates that the centrality measure is highly stable even when the sample is reduced, which means the ranking of node importance remains consistent. A CS-coefficient less than 0.25 suggests instability, implying that conclusions about node importance could change with different sample subsets. For interpretability, we strive for a CS-coefficient above 0.25 and, ideally, above 0.50, following the recommendations of Epskamp et al. (2018). For example, a CS-coefficient of 0.50 indicates that the remaining pairwise correlations among nodes can be maintained at 0.70 after dropping 50% of the sample (i.e., CS [cor = 0.7] = 0.50), suggesting adequate stability. Bootstrapped confidence intervals were used to investigate edge-weight accuracy, and bootstrapped difference tests were conducted to evaluate potential differences between edge weights and centrality within the same network (Epskamp et al., 2018).

#### Network comparison

The Network Comparison Test (NCT; van Borkulo et al., 2021) was utilized to examine gender differences in network structure, global strength, node strength, and edge weights (*γ* = 0.5, AND-rule, 1000 iterations). Specifically, *M* is the network invariance measure, typically examining the maximum difference in edge weights between two networks. A non-significant *M* value (i.e., the maximum edge weights difference being non-significant) indicates structural consistency between the estimated networks. The NCT also assesses global strength, which aggregates the weights of all these connections, indicating the overall degree of interconnectedness. The *S* measure is computed by summing the weights of all edges in the network and comparing these sums across groups. The same specified model parameters and bootstrapping procedures as in the overall sample were used to maximize parsimonious models, robust edge selection, and reliable estimates.

## Results

### Overall symptom structure

**Figure 1:**
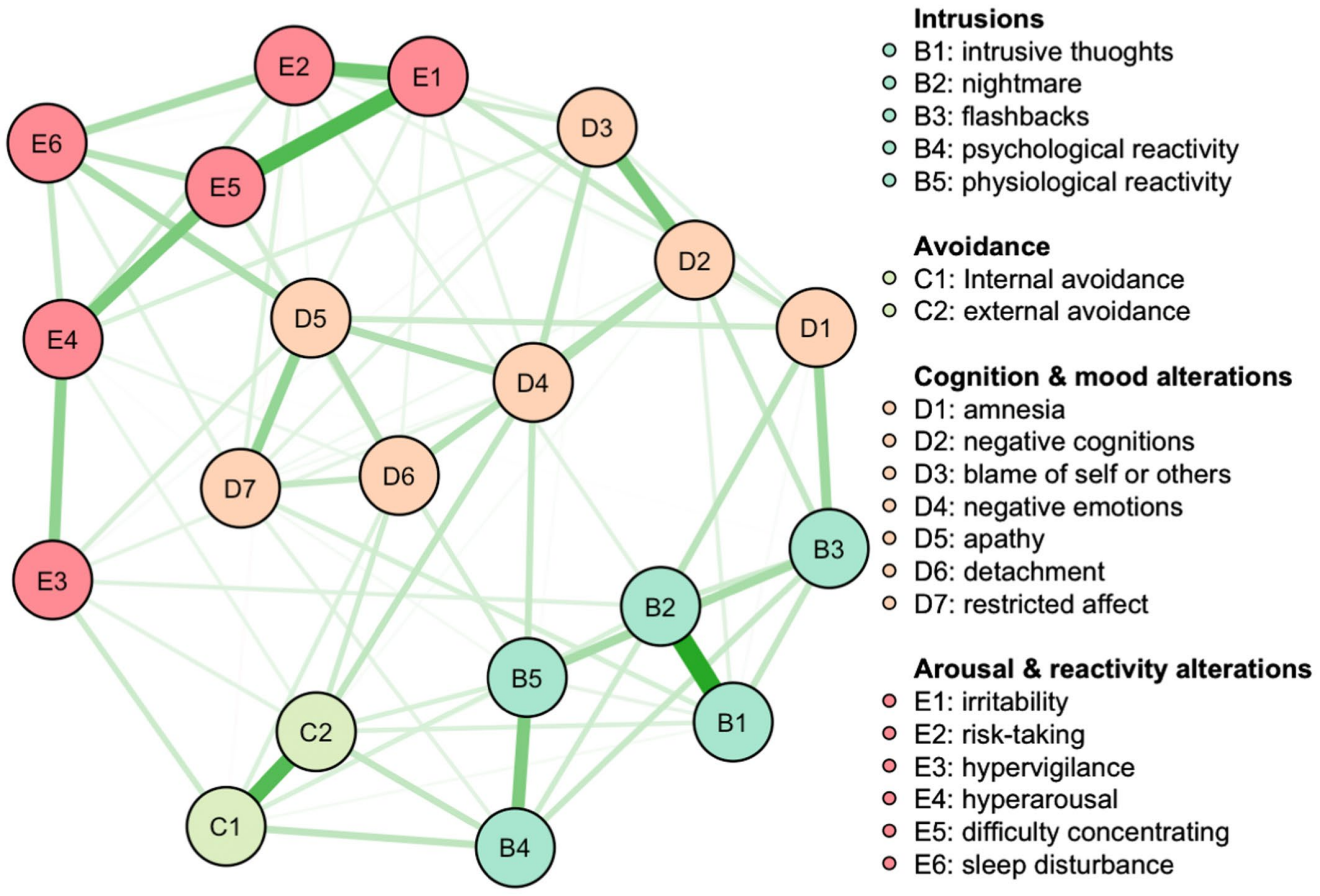
Full sample network. Nodes represent symptoms, edges partial correlations. Green edges indicate positive, red edges negative correlations. The larger the partial correlation, the thicker the edge.

The network structure for the overall sample (see Figure 1) indicated strong connections among Node B1 (intrusion) and Node B2 (nightmare), Node E1 (irritability) and Node E5 (difficulty concentrating), and Node C1 (internal avoidance) and Node C2 (external avoidance). Some nodes were not correlated, such as between Node B3 (flashbacks) and Node D4 (negative emotions).

#### Edge-weight accuracy

The presence of large bootstrapped CIs around the estimated edge weights in our network analysis necessitates cautious interpretations of the network’s structure. While it is clear that some edges, such as those linking intrusive thoughts with nightmares, irritability with difficulty concentrating, and internal with external avoidance, demonstrate strong connections due to non-overlapping CIs (see Supplementary Figure S1), the accuracy of other edges is less certain. Therefore, when considering the strong connections identified within the overall sample, readers should bear in mind that these are relative and not all connections are equally reliable or distinct from one another. The robustness of the connections should be interpreted in light of the overlapping CIs, with the understanding that the strength of some may be less definitive.

#### Centrality stability

When testing node centrality by examining consecutively smaller sub-samples, the stability of betweenness and closeness dropped steeply, while the stability of node strength and expected influence was more robust (Supplementary Figure S2). The CS-coefficient indicated that betweenness (CS [cor = 0.7] = 0.00) and closeness (CS [cor = 0.7] = 0.05) were not stable under subsetting cases. Node strength (CS [cor = 0.7] = 0.44) and expected influence (CS [cor = 0.7] = 0.60) performed better. As suggested by Epskamp et al. (2018), the CS-coefficients should be above 0.25 and preferably above 0.50. Thus, the orders of node *strength* and *expected influence* are reliable for interpretation, while the orders of betweenness and closeness are not. Only *strength* and *expected influence* centralities were considered in the discussion.

#### Centrality indices

**Figure 2:**
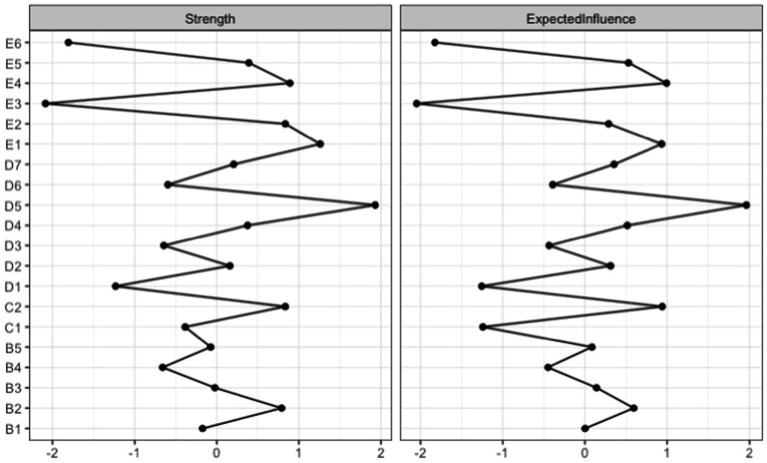
Standardized centrality indices for the PTSD symptoms of the full sample network.

Individual nodes were indexed according to the multiple metrics of importance, and the pattern for node importance was similar across different indices (see Figure 2). Node D5 (apathy) had the highest strength, followed by Node E1 (irritability), Node E4 (hyperarousal), C2 (external avoidance), B2 (nightmare), and E2 (risk-taking). Node D5 (apathy) had the highest expected influence, followed by Node E4 (hyperarousal), E1 (irritability), Node C2 (external avoidance), and E5 (difficulty concentrating).

#### Significance test

Many edges did not significantly differ from one another in terms of centrality (Supplementary Figure S3). Supplementary Figure S4 shows significant differences in strength from one another. Specifically, the strength of Node D5 (apathy) was significantly larger than some of other nodes, whereas the strength of Node E3 (hypervigilance) and of Node E6 (sleep disturbance) were significantly smaller than some of the other nodes. Supplementary Figure S5 shows significant differences in expected influence between nodes. Expected influence of Node D5 (apathy) was significantly larger than most of other nodes, whereas the expected influence of Nodes E3 (hypervigilance), E6 (sleep disturbance), and C1 (internal avoidance) was significantly smaller than most other nodes.

### Sex differences in symptom networks

#### Network comparison test

Quantitative comparisons of the global structure and strength of the male and female networks showed no statistically significant differences. An omnibus test of network structure invariance revealed that the global structures of the networks were not significantly different (*M* = 0.05, *p* = 0.82). Additionally, an omnibus test of network strength invariance showed that the global strengths of the networks were not different (*S* = 0.22, *p* = 0.89).

## Discussion

The current study aimed to model PTSD symptom structure among MSA survivors and to test whether symptom patterns may vary by sex. For the full sample, apathy, irritability, and hyperarousal appeared to be the most central symptoms as measured by the Strength index. As measured by Expected Influence index, apathy, hyperarousal, and external avoidance were the most central symptoms in this network. These findings are consistent with previous cross-sectional studies that have indicated the centrality of intrusion symptoms in the network structure of PTSD (Armour et al., 2017; Bryant et al., 2017) and childhood sexual trauma (Kratzer et al., 2022; McNally et al., 2017). The prominence of these symptoms suggests they have a critical role in the manifestation of PTSD symptoms following MSA.

The centrality indices provided information about the importance of individual symptoms within the network. Node D5 (apathy) exhibited the highest centrality across all indices, indicating its strong connections with the other symptoms in the network. This finding highlights the significance of apathy as a central symptom in the PTSD symptom network following MSA. Central symptoms are theorized to be potential treatment targets, in that changes in central symptoms could lead to changes in most symptoms in the network (Fried et al., 2017). However, such implications should be interpreted with great care since centrality measures only quantify partial correlations within this cross-sectional sample. As such, central symptoms are not necessarily the cause of other symptoms, and instead, apathy could arise as a consequence of other symptoms in the network. Apathy is also seen in depressive disorders, and research has shown PTSD treatments to be effective in improving depressive symptoms (Ronconi et al., 2015). Some research on Prolonged Exposure therapy (PE) for PTSD generally supports a reciprocal relation between PTSD and depressive symptoms, although this work showed stronger evidence that reductions in PTSD symptoms led to subsequent reductions in depressive symptoms compared to the other direction (Aderka et al., 2013; Brown et al., 2018; Peskin et al., 2019). However, a series of randomized control trials examining both PE and Cognitive Processing Therapy in military samples suggested that reductions in depressive symptoms may precede changes in PTSD symptoms (Bryan et al., 2024). Longitudinal designs are needed to examine whether reductions in apathy, in particular, would lead to reductions in other symptoms in the PTSD symptom network.

The current study also explored sex differences in PTSD symptom networks among male and female veterans and service members who experienced MSA. By applying a network approach, this study sought to understand the interconnections of PTSD symptoms to shed light on the potential sex differences in PTSD symptoms following MSA. Consistent with prior studies (e.g., Sexton et al., 2017; Tannahill et al., 2021), *t*-tests revealed comparable overall severity of PTSD symptoms among female and male MSA survivors. When comparing the network structures of PTSD symptoms between males and females, no statistically significant differences were found. Our results are congruent with previous PTSD symptom network studies examining sex differences (Armour et al., 2017; Birkeland et al., 2017; Gay et al., 2020), demonstrating that the symptom network structure of PTSD may be similar across sexes. Specifically, Gay and co-authors (2020) examined sex differences among a sample of natural disaster and accident victims, Armour et al. (2017) utilized a sample of US veterans, whereas Birkeland et al. (2017) recruited a sample of bombing victims, all of which observed that structures were similar. Thus, while rates of diagnosis may vary across sex, when females and males experience similar trauma types, there may be no sex differences in the structure or intercorrelations of symptoms. Our results add to this body of literature by demonstrating no significant sex differences in the network structure among a MSA exposed sample.

Furthermore, our findings suggest that the most central symptoms could be similar across sexes. Part of the appeal of network analysis has been the assumption that centrality may provide helpful treatment targets for those seeking care. This assumption should be considered with great care and in the context of the sample and network characteristics (e.g., Fried et al., 2017). Relative to other studies utilizing network analysis, our sample was more homogenous as it was composed of MSA survivors, and the PCL-5 was completed in reference to the MSA exposure.

Our results on edge-weight accuracy indicated notable variability in the estimated edge weights of PTSD symptoms, which highlights concerns raised within the field regarding the reliability and replicability of network analysis findings (Forbes et al., 2017, 2021). The large confidence intervals around edge weights observed in our analysis reflect the within-sample uncertainty. Sample size plays an important role in the replicability of network analysis, and our relatively small sample size could contribute to the uncertainty even though our sample is fairly homogeneous. As indicated in a recent meta-analysis (Isvoranu et al., 2021), different results observed across studies can be explained as a result of cross-study heterogeneity, sampling variation, and the performance of network estimation tools. The authors further stated that, despite large between-study heterogeneity, network models estimated from single samples can lead to network structures similar to the pooled network model. To enhance the reliability of our network findings and contribute to the ongoing methodological refinement, larger sample sizes and cross-validation methods should be employed (Epskamp et al., 2018). Such strategies could provide more definitive evidence for the robustness of network configurations and help determine the extent to which these models can be generalized across diverse PTSD populations.

While the current study sheds light on the symptom network structure of PTSD following MSA and the role of individual symptoms, it is important to interpret the results with caution due to certain limitations. The use of self-report measures, such as *PCL-5*, introduces the possibility of response bias and may limit the objectivity of symptom assessment. Future research could benefit from incorporating multiple assessment methods, including clinician-rated measures and biomarkers, to enhance the validity of the findings. Our study’s generalizability is constrained by an over-representation of White male officers, which may not fully capture the diverse experiences across different ranks and ethnic groups. Additionally, with only about 10% of our sample identifying as sexual minority, our ability to analyze PTSD symptoms across varied sexual orientations is limited. This demographic skew likely affects the applicability of our findings to the broader, increasingly diverse military population, suggesting a need for future research to include a more representative sample. Such studies could provide a deeper understanding of PTSD dynamics across different cultural, racial, and service backgrounds, which is essential for developing effective, culturally competent interventions. Additionally, the current study focused exclusively on MSA survivors among veterans and service members. Further investigations are warranted to validate and extend the current findings to other populations, such as civilian survivors of sexual trauma.

In conclusion, the present study utilized a network approach to model the symptom network structure of PTSD following MSA and explore potential sex differences in symptom patterns. Although no significant differences were observed between males and females in the network structure, the analysis identified key symptoms and their interconnections within the network.

The findings underscore the central role of intrusion symptoms, nightmares, irritability, difficulty concentrating, internal avoidance, and apathy in the PTSD symptom network following MSA. By unraveling the complex network of symptoms, this study provides insights that can inform future research, which would benefit from leveraging longitudinal designs and diverse samples to examine the temporal dynamics and generalizability of the findings.

## Data Availability

The raw data supporting the conclusions of this article will be made available by the authors on reasonable request, without undue reservation.
