# Supplementary material for: Similar PTSD symptom networks observed in male and female survivors of military sexual assault: implications for understanding trauma responses
Source: Front Psychol. 2024 Nov 7;15:1452417. doi: 10.3389/fpsyg.2024.1452417 (PMC11580701; doi:10.3389/fpsyg.2024.1452417)
Supplement: Supplementary file 1 [file Table_1.docx]

**Figure S1.** Bootstrap 95% confidence intervals for estimated edge weights for the symptom network for the full sample


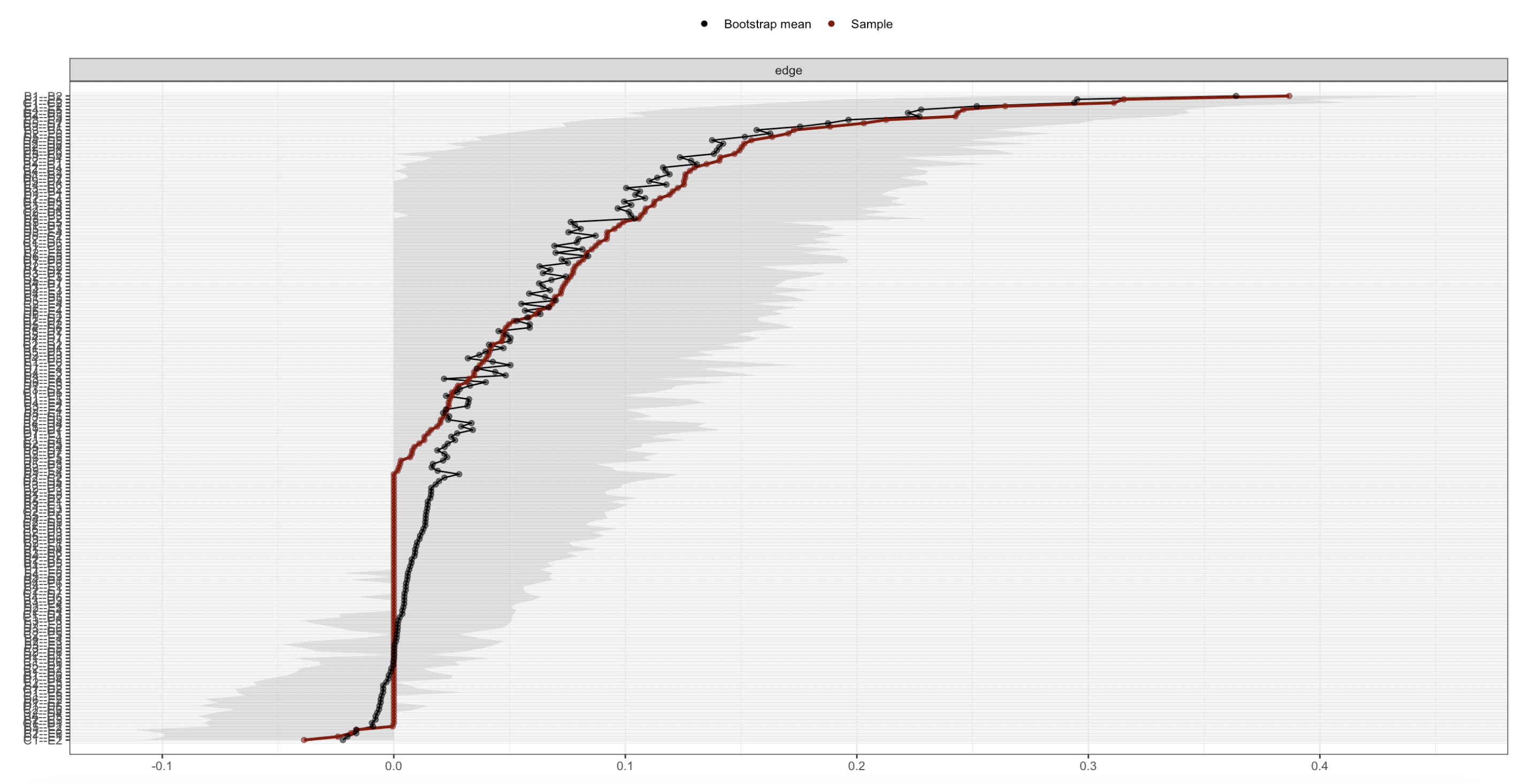


Each horizontal line represents one edge. Edge weights are represented by the red line. The 95% confidence intervals are indicated by the grey area.

**Figure S2.** The average correlation between bootstrap centrality measures of networks sampled with case-dropping and the symptom network for the full sample.


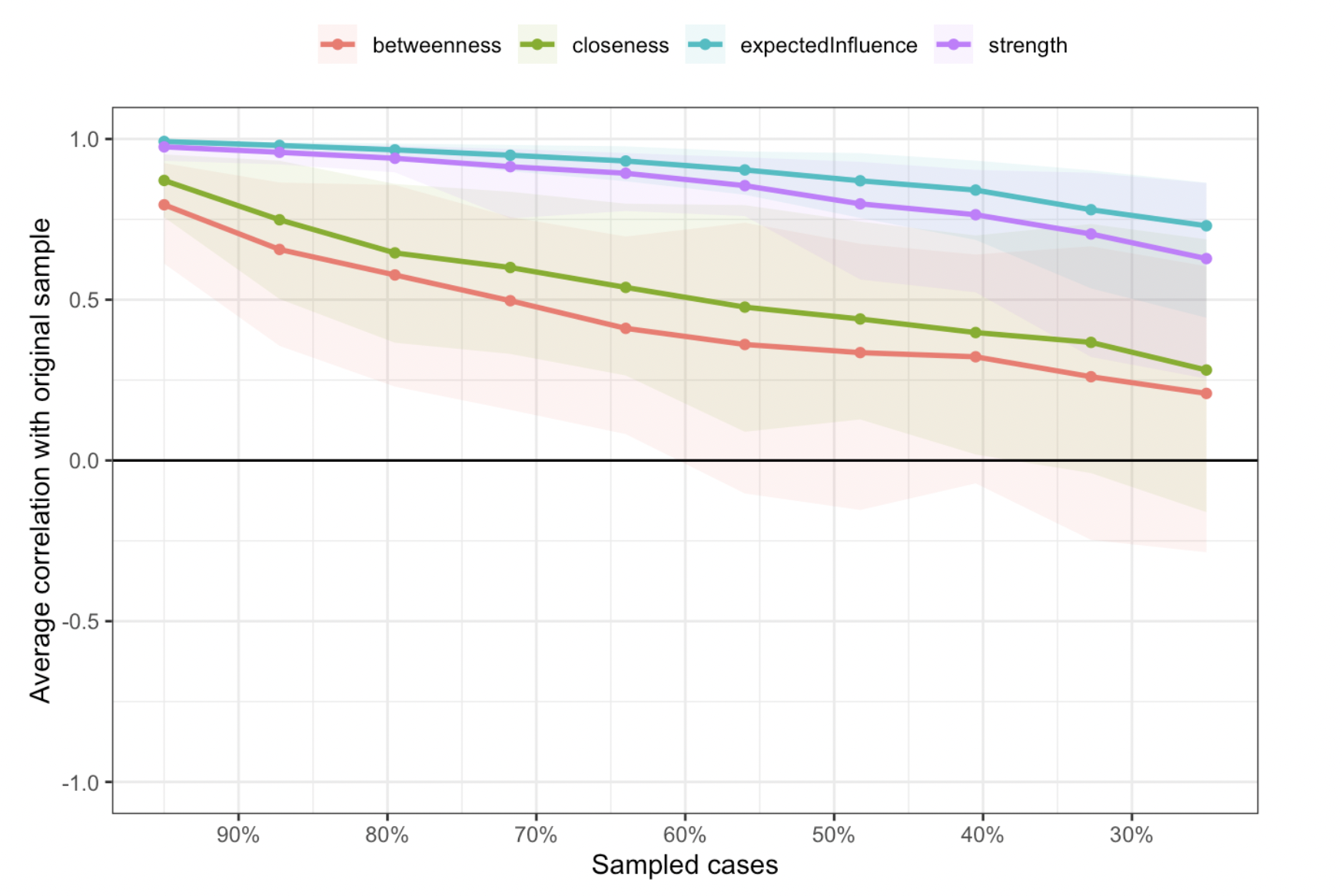


**Figure S3.** Bootstrap edge weights difference test between non-zero estimated edge-weights in the network of PTSD symptoms in the full sample.


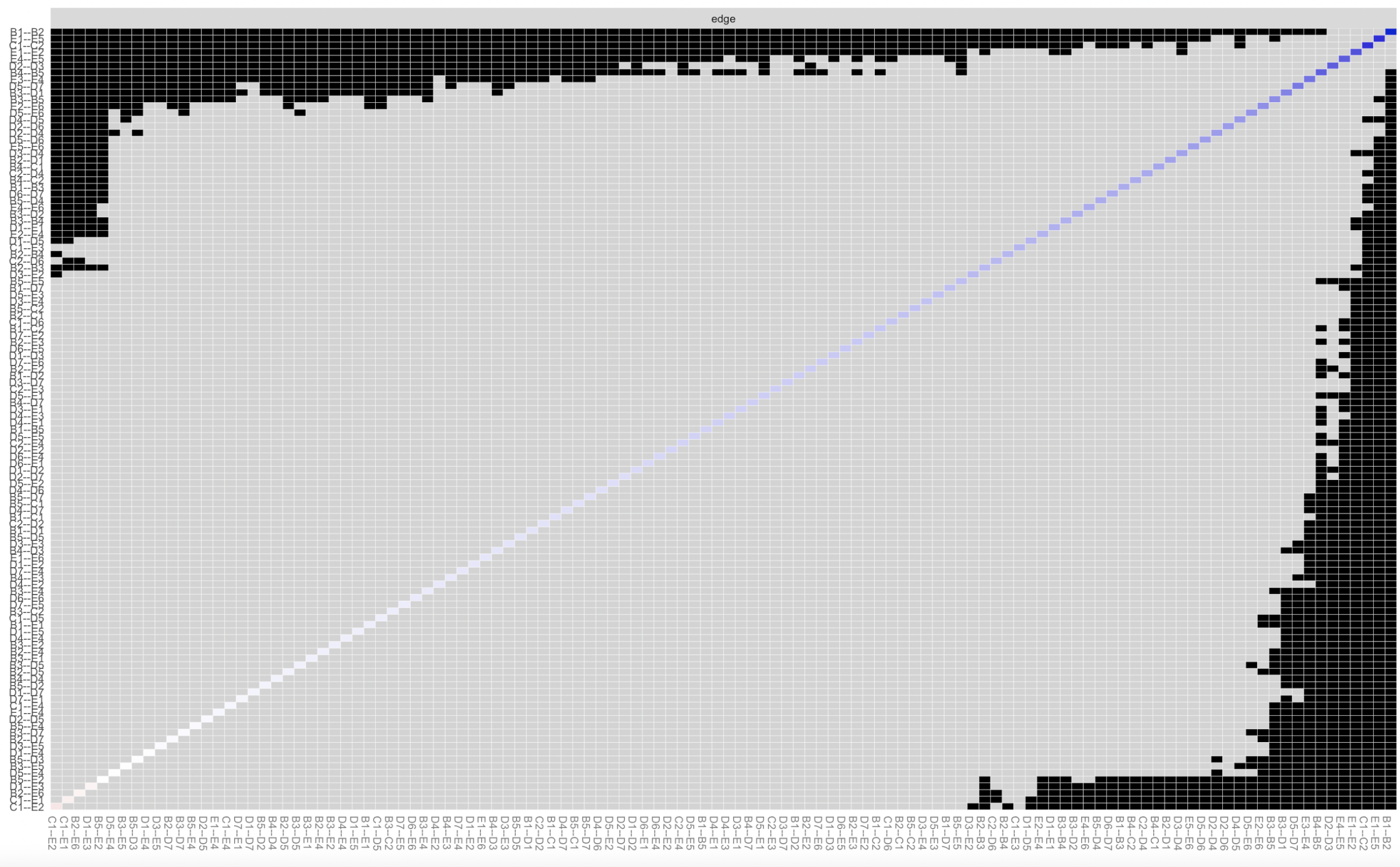


Bootstrapped difference tests (α = 0.05) between edge-weights in the networks of PTSD symptoms for the full sample. Significant differences between two edges are indicated by black boxes, non-significant differences are indicated by grey boxes. The colour of the boxes (ranging from white to blue) corresponds to the thickness of the edge.

**Figure S4.** Bootstrap node strength difference test between nodes of the PTSD symptom structure of the full sample.


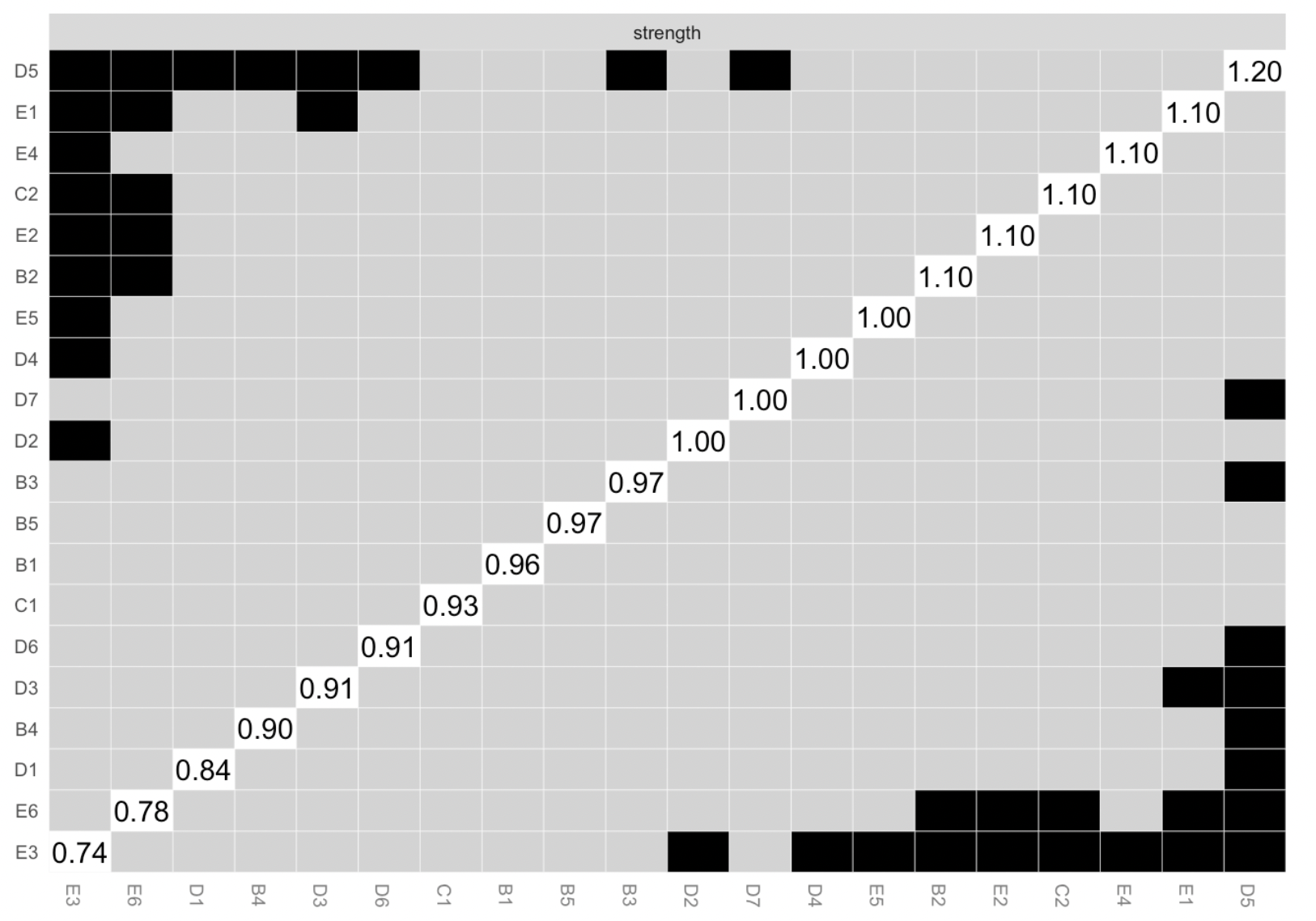


Significant differences between two edges are indicated by black boxes, non-significant differences are indicated by grey boxes. The value of node strength is indicated by the number in the white boxes.

**Figure S5.** Bootstrap node expected influence difference test between nodes of the PTSD symptom structure of the full sample.


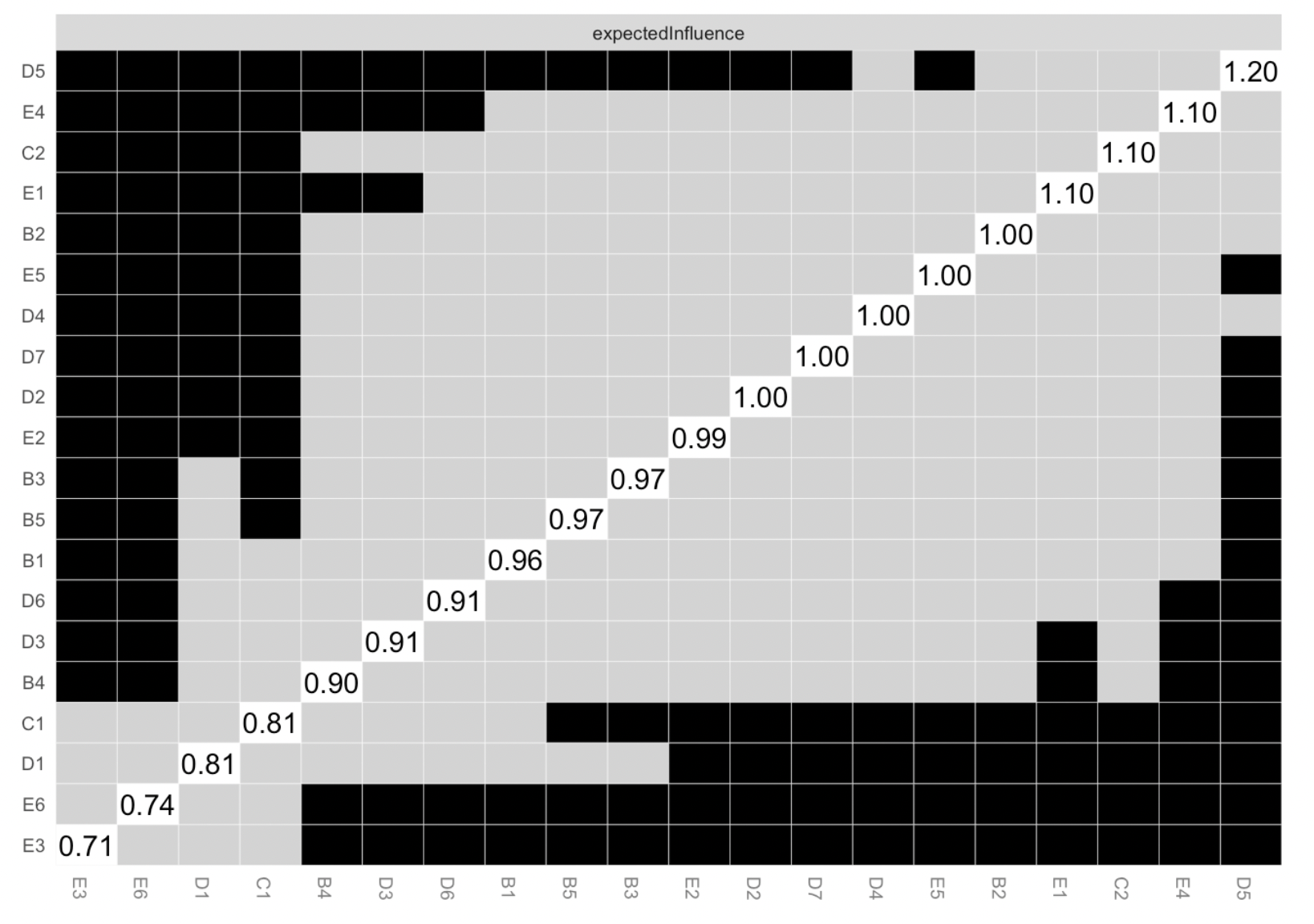
 Significant differences between two edges are indicated by black boxes, non-significant differences are indicated by grey boxes. The value of node strength is indicated by the number in the white boxes.
